# Galectin-1 Inhibition as a Strategy for Malignant Peripheral Nerve Sheath Tumor Treatment

**DOI:** 10.3390/cells14070515

**Published:** 2025-03-31

**Authors:** Hsiao-Chi Wang, Keila E. Torres, Roger Xia, Marcio H. Malogolowkin, Ssu-Wei Hsu, Ching-Hsien Chen, Tsung-Chieh Shih

**Affiliations:** 1 Department of Research and Development, Kibio Inc., Houston, TX 77021, USA; 2 Department of Surgical Oncology, The University of Texas MD Anderson Cancer Center, Houston, TX 77030, USA; 3 Department of Biomedical Data Science, Stanford University, Stanford, CA 94304, USA; 4 Division of Pediatric Hematology-Oncology, UC Davis Children’s Hospital, Sacramento, CA 95816, USA; 5 Comprehensive Cancer Center, University of California at Davis, Davis, CA 95616, USA; 6 Divisions of Nephrology and Pulmonary, Critical Care and Sleep Medicine, Department of Internal Medicine, University of California at Davis, Davis, CA 95616, USA; 7 Department of Translational Molecular Pathology, The University of Texas MD Anderson Cancer Center, Houston, TX 77030, USA

**Keywords:** MPNST, Galectin-1, RAS, LLS30, EMT

## Abstract

Neurofibromatosis type 1 (NF1) is an inherited disorder that predisposes individuals to malignant peripheral nerve sheath tumors (MPNSTs), a highly aggressive sarcoma with limited treatment options and poor prognosis. This study explores the potential of targeting the interaction between Galectin-1 and Ras as a novel therapeutic strategy for MPNSTs. Through molecular docking, we identified critical residues involved in the Galectin-1 and H-Ras interaction. We developed LLS30, a compound designed to target this Ras-binding pocket on Galectin-1, and tested its efficacy. LLS30 effectively disrupted the Galectin-1/Ras interaction, causing Ras delocalization from the plasma membrane and inhibiting Ras signaling. In vitro experiments showed that LLS30 significantly decreased MPNST cell proliferation and induced apoptosis. In vivo, LLS30 demonstrated potent anti-tumor effects, reducing tumor size, inhibiting metastasis, and extending survival in animal models. Transcriptome analysis further revealed the downregulation of KRAS signaling and inhibition of pathways associated with epithelial–mesenchymal transition. These findings suggest that targeting Galectin-1 with LLS30 offers therapeutic potential for MPNSTs and could be beneficial for other cancers driven by Galectin-1 and Ras signaling.

## 1. Introduction

Neurofibromatosis type 1 (NF1) is a tumor that grows along nerves in the skin, brain, and other parts of the body. NF1 is the most common inherited neurological disorder, affecting approximately 1 in 3000 people throughout the world [1]. The most common tumors associated with NF1 are neurofibromas, which are generally non-cancerous and are categorized into dermal and plexiform types [2,3]. Although neurofibromas are typically benign, plexiform neurofibromas have the potential to develop into malignant peripheral nerve sheath tumors (MPNSTs), also referred to as malignant schwannomas or neurofibrosarcomas. Between 8% and 13% of people with NF1 will get an MPNST in their lifetime, so approximately 800,000 people worldwide are affected per year [4,5,6]. Approximately 20% of cases are diagnosed in the pediatric population [7].

An MPNST is considered a high-grade sarcoma with the potential to recur and metastasize. Currently, surgical resection is the main treatment for this disorder [8]. Unfortunately, complete surgical removal is almost impossible because tumors may develop in deep peripheral nerves or roots. Surgical resection can even cause disability. MPNSTs pose significant management challenges. For unresectable or metastatic diseases, chemotherapeutic drugs are only marginally effective (with a response rate of <21%), and initial responses to therapy are usually short-lived with a recurrence rate of 40–65%, followed by rapid progression and death [9]. As such, 5-year overall survival rates remain low (the 5-year survival is <50%) [10,11]. However, there are no effective systemic therapies for MPNST patients.

NF1 is a hereditary condition caused by mutations in the *NF1* gene, which encodes neurofibromin. Neurofibromin plays a role as a Ras GTPase-activating protein (GAP), converting active Ras-GTP to inactive Ras-GDP [12]. By promoting the hydrolysis of GTP to GDP, neurofibromin serves as an inhibitor of Ras activity [13]. Mutations in the *NF1* gene in neurofibromas and MPNSTs lead to higher levels of active Ras-GTP [14,15]. Ras is initially synthesized in its inactive form and associated with the cell membrane after undergoing modifications such as prenylation and palmitoylation. It becomes further stabilized by interacting with chaperone proteins, such as PDEδ and RAP1GDS [16,17]. The presence of Ras at the membrane is essential for triggering signaling cascades involved in oncogenesis. Thus, targeting Ras-binding proteins to disrupt its membrane association offers significant therapeutic potential for inhibiting Ras-driven cancer progression, especially in MPNSTs.

Galectin-1 (Gal-1), a 14 kDa lectin, is one of a galectin family with an affinity for β-galactosides. The 135 kDa Gal-1 protein is encoded by the gene *LGALS1* at 22q13.1. Increased Gal-1 expression by tumor and connective tissue is regarded as a sign of malignant progression and often correlates with aggressiveness and a metastatic phenotype [18,19,20]. Several proteins that interact with Gal-1 have been identified, including H-Ras, integrins, laminins, fibronectin, vitronectin, osteopontin, neuropilin-1, CD44, CD146, and CD326, among others [21,22]. Our previous study has shown elevated Gal-1 levels in MPNST patients and cells, with Gal-1 knockdown leading to Ras pathway suppression and inhibition of cancer cell proliferation both in vitro and in vivo [23]. Mechanistically, Gal-1, functioning as a chaperone for Ras, interacts and stabilizes activated Ras at the plasma membrane, thereby resulting in the activation of the Ras oncogenic signaling pathway [23]. Disruption of Gal-1/H-Ras(G12V) interaction could be an effective strategy for treating MPNSTs. However, the exact residues that participate in the interaction between Gal-1 and H-Ras(G12V) remain unclear.

This study introduces a novel therapeutic strategy for MPNSTs by targeting the Gal-1 and Ras interaction, a pathway previously unexamined in this cancer type. We pinpointed critical residues responsible for the Gal-1/H-Ras(G12V) interaction and showed that LLS30, a Gal-1 inhibitor, targets the Ras-binding site on Gal-1, blocking the interaction and inhibiting tumor growth. In addition, LLS30 was observed to impact the EMT pathway, preventing metastasis. These findings provide a strong foundation for advancing LLS30 as a potential treatment for aggressive cancers driven by Gal-1/Ras signaling.

## 2. Materials and Methods

### 2.1. Cell Lines

The cell lines used in this study include MPNST-derived cell lines NF02.2 and NF96.2, as well as HEK293T. These cells were cultured in DMEM containing 10% fetal bovine serum and 1% penicillin/streptomycin, and maintained in a 5% CO_2_ atmosphere at 37 °C. Mycoplasma contamination was tested monthly as part of routine quality control.

### 2.2. Cell Viability and Apoptosis Assay

To evaluate cell viability, 5 × 10^3^ NF96.2 and NF2.2 cells were plated in 96-well plates and allowed to adhere for 24 h before treatment with LLS30 or OTX008 for 72 h. After the incubation, the medium was removed, and cells were treated with the designated concentrations of LLS30 or OTX008. Stock solutions of LLS30 and OTX008 (10 mM) were initially dissolved in 100% DMSO. A 100 µM working solution of LLS30 was prepared by diluting the stock 1:100 in a cell culture medium and further serially diluted. Cell viability was assessed using the MTT assay at specified time points. For apoptosis detection, caspase-3/7 activity was measured using the luminescent caspase-Glo 3/7 assay kit (Promega, Madison, WI, USA) after 12 and 24 h of treatment with 5 μM LLS30 or 0.05% DMSO.

### 2.3. Immunoblotting Analysis

The immunoblotting procedure was performed according to a previously described method [23]. In brief, cells were lysed in RIPA buffer and incubated on ice for 20 min. After centrifugation, the total cell lysates were collected and protein concentrations were determined using a BCA assay. Equal amounts of lysates (20 μg) were mixed with 2X Laemmli SDS-PAGE sample buffer, heated, separated on 12% SDS-PAGE gels, and transferred to PVDF membranes. Membranes were blocked with 10% non-fat dry milk in Tris-buffered saline and incubated overnight at 4 °C with primary antibodies targeting phospho-Erk (Thr202/Tyr204, Cell Signaling, Danvers, MA, USA), Erk (Cell Signaling), or beta-actin (Cell Signaling). After washing with TBS-T, membranes were incubated with HRP-conjugated secondary antibodies at 37 °C for 1 h. Chemiluminescence detection was performed using an ECL substrate and captured with a CCD camera.

### 2.4. Protein–Protein Docking Simulation

The 3D structures of the proteins H-Ras(G12V) and Gal-1 were downloaded from the RCSB PDB Database (https://www1.rcsb.org/, accessed on 13 April 2022), with the PDB ID for H-Ras(G12V) being 4EFM and for Gal-1 being 6F83. Protein–protein docking in ClusPro1 was utilized for molecular docking simulation and predicting the binding affinity of H-Ras(G12V) with Gal-1. Gal-1 was set as the ligand and H-Ras(G12V) as the receptor for protein docking. The ligand underwent 70,000 rotations, with translations along the x, y, and z axes relative to the receptor on a grid during each rotation. The translation yielding the best score from each rotation was selected. From an initial set of 70,000 rotations, 1000 rotation/translation combinations with the lowest docking scores were selected. These 1000 ligand positions were then grouped using a greedy clustering algorithm with a 9 Å C-alpha RMSD radius. The goal was to identify ligand positions with the highest number of neighboring ligands, which were likely to represent more stable binding modes. From these clusters, the top ten cluster centers with the greatest number of cluster members were retrieved for detailed inspection. To further assess the docking accuracy, intermolecular contacts from the most probable binding poses were analyzed. Key binding interactions were experimentally validated to confirm the docking predictions. This validation step ensured that the identified binding poses accurately represented the interaction between the ligand and the target protein. Docked structures and the interface residues were analyzed using the MOE2 (Molecular Operating Environment) software suite (version 2022 02, Chemical Computing Group, Montreal, QC, Canada). Molecular graphics were visualized using PyMOL (version 2.5.2, Schrödinger, Mannheim, Germany) for better structural interpretation.

### 2.5. Plasmid Construction and Protein Production and Purification

The pcDNA3.1/His-Gal-1 plasmid was commercially purchased from Gene Script (Piscataway, NJ, USA), with sequences encoding Gal-1 proteins based on GenBank (NM_002305). Inverse PCR Mutagenesis was used to introduce mutation D123A in previously cloned sequences with the Phusion Site-Directed Mutagenesis Kit (ThermoScientific, Waltham, MA, USA). The presence of the correct mutation was confirmed via sequencing. HEK293T cells were transiently transfected with the plasmids pcDNA3.1/His-Gal-1 and pcDNA3.1/His-Gal-1 D123A using Lipofectamine 2000 transfection reagent (Life Technologies, Carlsbad, CA, USA) according to the manufacturer’s protocol. After 5 days post-transfection, the cell pellet was collected by centrifugation at 400× *g* for 5 min, and subsequently, Gal-1 wild type (WT) and Gal-1 D123A mutant (Mut) proteins were purified using ProBond™ Nickel-Chelating Resin (ThermoScientific, Waltham, MA, USA), as per the manufacturer’s instructions. The purity of the obtained fraction was assessed by SDS-PAGE analysis, with quantification performed using the Bradford method.

### 2.6. Pull-Down Assay

An amount of 50 μL of streptavidin-agarose gel slurry (ThermoScientific, Waltham, MA, USA) was mixed with 50 μg of biotinylated LLS30 in a spin column and incubated for 4 h at 4 °C. The Sepharose slurry was then added to 20 μg of whole cell lysate (WCL) from NF96.2 cells or 2 μg of Gal-1 WT or Gal-1 Mut and incubated with rotary agitation overnight at 4 °C. After incubation, the Sepharose was washed with PBS, and bound proteins were eluted using 150 mM glycine, pH 2.5, for 10 min. The eluent was neutralized by adding 10 μL of neutralization buffer (Tris, pH 8.0) and subsequently analyzed by immunoblotting to detect Gal-1.

### 2.7. Co-Immunoprecipitation Assay

A total of 2 × 10^6^ NF96.2 cells were treated overnight with either 2 µM LLS30 or 0.02% DMSO, followed by membrane protein extraction using the ProteoExtract^®^ Native Membrane Protein Extraction Kit (MilliporeSigma, Burlington, MA, USA). For immunoprecipitation, 10 µL of anti-Gal-1 antibody (Abcam, Cambridge, UK) was mixed with 100 µL of Protein A/G Sepharose slurry (Abcam) and incubated for 4 h at 4 °C. The Sepharose slurry was then added to the protein mixtures and rotated overnight at 4 °C. After washing the Sepharose with PBS, bound proteins were eluted using 150 mM glycine (pH 2.5) for 10 min. The eluent was neutralized with 10 mL of neutralization buffer (Tris, pH 8.0) and analyzed by immunoblotting to assess Ras levels.

### 2.8. In Vivo Animal Assays

The animal experiments in this study were approved by the Institutional Animal Care and Use Committee (IACUC) at the campus. LLS30 stock solutions (6×) were prepared by dissolving 50% absolute alcohol and 50% Tween 80 to achieve a concentration of 15 mg/mL, which was then diluted with saline to a working solution of 2.5 mg/mL. Male athymic BALB/c nude (nu/nu) mice, obtained from the Jackson Laboratory, were used in this study. For the MPNST orthotopic xenograft mouse models, the mice were anesthetized with isoflurane, and bilateral exposure of the sciatic nerves was performed at mid-thigh. A cell suspension (5 × 10^5^ cells in 5 μL) was injected into the sciatic nerve using a Microliter Syringe Model 701 N (Hamilton Company, Reno, NV, USA). The surgical site was sealed with tissue adhesive, and the mice were returned to pathogen-free housing for recovery. The tumors became palpable after 6 weeks. The mice were randomly assigned to two groups (N = 6) and then intraperitoneally administered (1) a vehicle (8.7% alcohol/8.7% Tween 80) or (2) 10 mg/kg of LLS30 daily via intravenous injection for 14 consecutive days. After two weeks, bioluminescence signals were measured using the IVIS 200 Imaging System (version 2.50, Caliper LifeSciences, Hopkinton, MA, USA) following intraperitoneal injection of 100 mg/kg of D-luciferin. Tumor signal quantification was performed using Aura software (version is 1.07.84_V2, Aura, Boston, MA, USA) and the mice were subsequently sacrificed, with tumors excised for the activated Ras assay. For the lung metastasis model, 1 × 10^6^ luciferase-tagged NF96.2 cells were injected intravenously into the mice, followed by LLS30 treatment (5 mg/kg once daily for 5 days) two weeks later. Eight weeks post-implantation, bioluminescence signals were again detected using the IVIS 200 Imaging System, following intraperitoneal injection of 100 mg/kg of D-luciferin. Randomization and blinding were employed throughout the experiments to ensure unbiased data collection.

### 2.9. Ras Activation Assay

Activated Ras was measured using the Ras activation assay kit (MilliporeSigma, Burlington, MA, USA), following the manufacturer’s protocol. Briefly, xenograft tumors were excised and homogenized, and 200 µg of tissue lysates were incubated with Raf-1 Ras-binding domain (RBD)-agarose beads for 30 min at 4 °C. After centrifugation at 14,000× *g* for 10 s at 4 °C and washing, the agarose-bound Ras was mixed with 2× Laemmli reducing sample buffer (126 mM Tris/HCl, 20% glycerol, 4% SDS, 0.02% bromophenol blue). The samples were then separated by SDS-PAGE and analyzed by immunoblotting using an anti-Ras antibody (MilliporeSigma, Burlington, MA, USA).

### 2.10. Transcriptome Sequencing and Enrichment Analysis

Total RNA was extracted from the control and LLS30-treated NF96.2 cells using the PureLink RNA Mini Kit (Invitrogen, Waltham, MA, USA) according to the manufacturer’s protocol. RNA quality was assessed using the Agilent 2100 Bioanalyzer (Agilent Technologies, Santa Clara, CA, USA). mRNA sequencing libraries were prepared and sequenced in the paired-end format using the Illumina HiSeq 4000 platform (Illumina, San Diego, CA, USA). Significant differentially expressed genes (DEGs) were identified using a 1.5-fold change threshold and a significance level of *p* < 0.05. For normalization, raw RNA-seq data were processed using the DESeq2 package to account for sequencing depth and library size. The data were normalized to ensure comparability across samples. Volcano plots were generated using Sigmaplot software (version 14.0, Sigmaplot, San Jose, CA, USA) and Hallmark gene set enrichment analysis was conducted via the Enrichr website (https://maayanlab.cloud/Enrichr/, accessed on 28 November, 2024.) using gene symbols.

### 2.11. Gene Set Enrichment Analysis (GSEA)

GSEA was performed using the Java desktop software (http://software.broadinstitute.org/gsea/index.jsp, accessed on 27 February 2022), following previously established protocols [24]. Genes were ranked according to shrunken limma log2 fold changes, and the GSEA tool was run in “pre-ranked” mode with all default settings.

### 2.12. Statistics Analysis

In vitro experiments were performed in triplicate across two independent trials, with the results presented as the mean ± SD. Survival curves for both the control and LLS30 treatment groups were generated using the Kaplan–Meier method, and statistical comparisons were made using the log-rank test. The Student’s *t*-test (two-tailed) was applied to compare datasets between two groups with similar variance. A *p*-value of < 0.05 was considered statistically significant. Statistical differences, when compared with the controls, are denoted as * (*p* < 0.05), ** (*p* < 0.01), or *** (*p* < 0.001).

## 3. Results

### 3.1. Molecular Dynamics Simulation of Gal-1, Ras, and LLS30 Interactions

**Figure 1:**
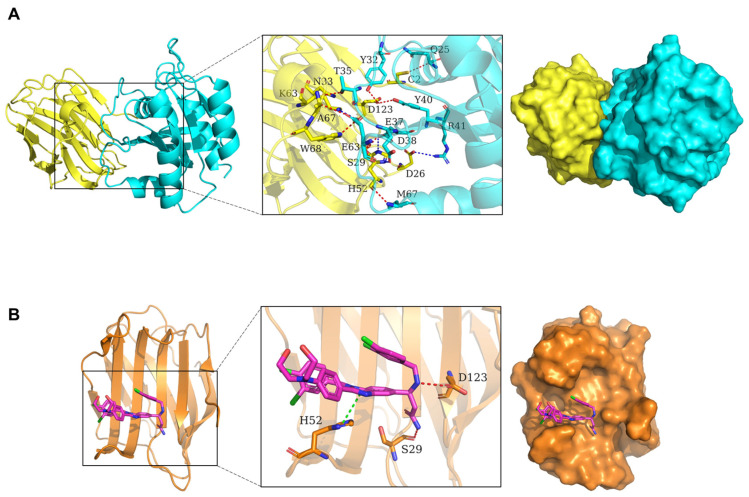
Molecular dynamics simulation of Gal-1, Ras, and LLS30 interactions. (**A**) The 3D binding model of Gal-1 with H-RAS(G12V). Gal-1 is colored with yellow; H-Ras(G12V) is colored with cyan. The residues in Gal-1 are represented as yellow sticks, and the residues in H-Ras(G12V) are shown as cyan sticks. Red dashes indicate hydrogen bond interactions, while blue dashes represent salt bridges. (**B**) The 3D binding model of Gal-1 with LLS30 is shown, where Gal-1 is colored orange and LLS30 is magenta. The residues in Gal-1 are depicted as orange sticks. Red dashes indicate hydrogen bond interactions, and green dashes represent Pi-Pi interactions.

Both our studies and others have indicated that Gal-1 functions as a chaperone for Ras and is necessary for Ras membrane localization, which is the primary site for the activation of Ras [23,25]. However, the specific residues for this interaction remain unclear. To investigate the binding mode of H-Ras(G12V) with Gal-1, docking simulation studies were carried out. The interaction between H-Ras(G12V) with Gal-1 is shown in Figure 1A. The contact list between H-Ras(G12V) with Gal-1 is shown in Table 1. Docking simulation studies indicate that the residues Gln25, Tyr32, Thr35, Asp38, Tyr40, Glu63, and Met67 in H-Ras(G12V) are involved in binding with Cys2, Asp123, Asn33, Ser29, Ala67, Trp68, and His52 in Gal-1 through hydrogen bond interactions. The residues Glu37, Asp38, Arg41, and Glu63 in H-Ras(G12V) are involved in binding with His52, Asp26, and Lys63 through salt bridges. Given the critical role of Gal-1 in Ras activation, our objective was to disrupt the Gal-1/Ras interaction as a potential therapeutic strategy. Targeting the Ras-binding pocket on Gal-1 may effectively block the interaction between Gal-1 and Ras. To explore this possibility, we tested LLS30 in this study to assess its ability to bind to the same pocket on Gal-1 where H-Ras(G12V) interacts. Molecular docking studies revealed that the aromatic groups of LLS30 are positioned within the hydrophobic core of Gal-1’s binding pocket, where residues His52, Ser29, and Asp123 play crucial roles in interacting with LLS30 through Pi-Pi stacking and hydrogen bonding (Figure 1B). These findings from the molecular docking studies indicate that LLS30 has the potential to interfere with the Gal-1/Ras interaction by binding to key residues within Gal-1’s binding pocket.

### 3.2. LLS30 Disrupts Gal-1/Ras Interactions and Resulting Delocalization of RAS

**Figure 2:**
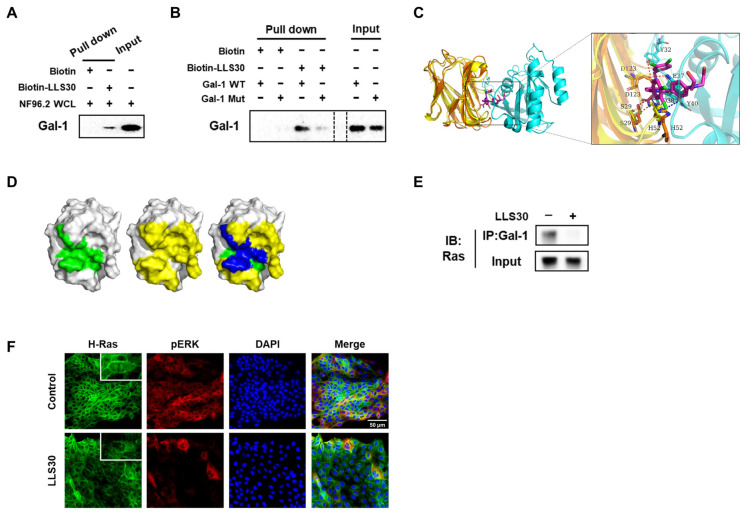
LLS30 interacts with Gal-1 and disrupts Gal-1/Ras interactions in MPNST cells. (**A**) The pull-down assay confirmed the direct interaction between LLS30 and Gal-1 from NF96.2 whole cell lysate (WCL). Biotin–LLS30 conjugated with streptavidin-agarose beads was incubated with NF96.2 WCL to pull down interacting proteins. After thorough washing to remove non-specifically bound proteins, the proteins bound to the beads were eluted and confirmed by immunoblots using an antibody against Gal-1. The input lane represents NF96.2 WCL without pull-down, serving as an input control to show the baseline level of Gal-1 in the cells. (**B**) LLS30 pulls down Gal-1 wild type (WT) but exhibits reduced binding with Gal-1 mutant (D123A) (Mut). Biotin–LLS30 bound to streptavidin-agarose beads was incubated with recombinant proteins Gal-1 WT or Mut. After washing, the level of bound protein was detected via immunoblots with an anti-Gal-1 antibody. Gal-1 WT or Mut alone was loaded as input. (**C**) The superposition diagrams of the H-Ras(G12V)-Gal-1 complex and the Gal-1-LLS30 (magenta) complexes. (**D**) The binding region of LLS30 on the surface of the Gal-1 protein is shown in green (left), while the binding region of H-Ras(G12V) on the Gal-1 protein surface is depicted in yellow (middle); and the overlapping region where LLS30 and H-Ras(G12V) bind on the Gal-1 protein surface is highlighted in blue (right). (**E**) Co-immunoprecipitation experiments were conducted to examine the interaction between Gal-1 and H-Ras following treatment with either LLS30 or the vehicle DMSO. F96.2 cells were first treated overnight with either LLS30 or DMSO. Subsequently, cell membrane proteins were extracted, followed by co-immunoprecipitation using an anti-Gal-1 antibody. The input consisted of total membrane protein extracted from NF96.2 cells without co-IP. The eluted proteins from co-IP and the total membrane proteins (without co-IP) were analyzed using SDS-PAGE and immunoblotted with an anti-Ras antibody. (**F**) Immunofluorescence microscopy demonstrating the expression and localization of H-Ras and pERK in NF96.2 cells.

To validate the findings of the docking studies, we performed pull-down assays to verify the physical interaction with Gal-1. Streptavidin-agarose bead-bound biotin–LLS30 was prepared and incubated with NF96.2 WCL to pull down interacting proteins. The proteins bound to the target were eluted, and then analyzed using SDS-PAGE, followed by immunoblotting with an anti-Gal-1 antibody. The band that appeared on the immunoblot indicated the presence of Gal-1 in the WCL pulled down by the biotin–LLS30 complex (Figure 2A), confirming that Gal-1 interacts with LLS30. Furthermore, given that Asp123 plays a significant role in the binding of LLS30 to Gal-1 through hydrogen bond interactions, we introduced a single point mutation (D123A) in Gal-1 to test its importance. The results showed that the D123A mutation reduced binding affinity (Figure 2B), confirming Asp123’s critical role in the interaction. These findings confirm LLS30’s ability to bind to Gal-1 in MPNST cells, suggesting its potential as a Gal-1 inhibitor for therapeutic application in MPNSTs. Further binding model analysis showed that both H-Ras(G12V) and LLS30 can interact with residues His52, Ser29, and Asp123 on Gal-1 (Figure 2C,D). These findings suggested that LLS30 may interfere with the Gal-1/Ras interaction. To verify this, co-immunoprecipitation (co-IP) was performed to assess whether LLS30 affects Ras binding to Gal-1. The results showed a noticeable decrease in the amount of Ras bound to Gal-1 in the presence of LLS30 (Figure 2E). Importantly, LLS30 treatment was found to disrupt the association of RAS with the plasma membrane (Figure 2F). Moreover, phosphorylation of the ERK was found to be significantly reduced after LLS30 treatment (Figure 2F). Collectively, these results demonstrate that LLS30 targets Gal-1, disrupts its interaction with Ras, leads to Ras dissociation from the plasma membrane, and suppresses the Ras/Erk signaling pathway in MPNST cells.

### 3.3. Anti-Cancer Activity of LLS30 Against MPNST Cells In Vitro

**Figure 3:**
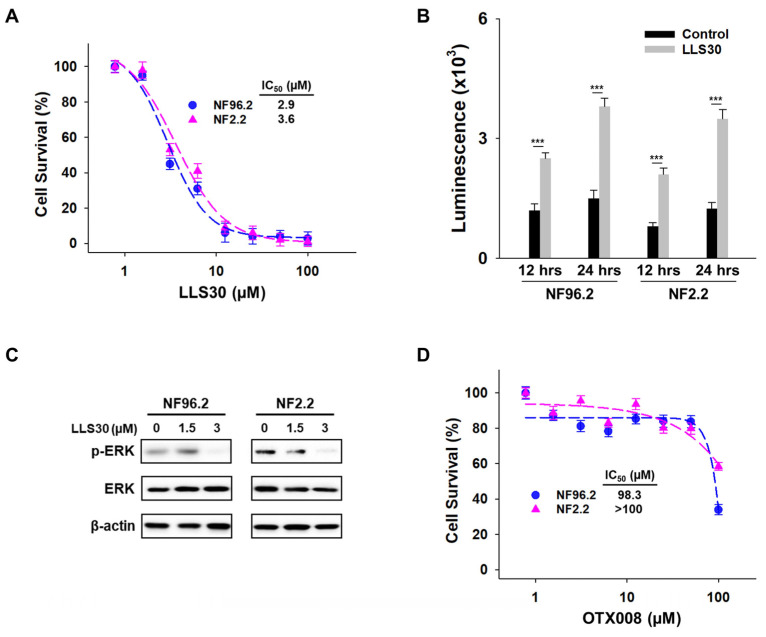
Effects of LLS30 on MPNST cells. (**A**) Cell viability of NF96.2 and NF2.2 MPNST cells following treatment with different concentrations of LLS30 for 72 h. The IC_50_ values for LLS30 in NF96.2 and NF2.2 cells are 2.9 μM and 3.6 μM, respectively. (**B**) Caspase-3/7 activity in NF96.2 and NF2.2 cells following 12 and 24 h of treatment with 0.05% DMSO or 5 µM LLS30. (**C**) Immunoblots of phospho-Erk, Erk, and β-actin in NF96.2 and NF2.2 treated with 0, 1.5, or 3 μM of LLS30 for 24 h. (**D**) Effect of OTX008 on MPNST cell lines. Cell viability of NF96.2 and NF2.2 MPNST cells was assessed after treatment with indicated concentrations of OTX008 for 72 h. *** *p* < 0.001; two-tailed Student’s *t*-test. Data shown are mean ± s.d.

Given that LLS30 disrupts Gal-1/Ras interactions, we further evaluated the potential anti-cancer effects of LLS30 on MPNST cells. Proliferation assays showed a significant reduction in MPNST cell growth in a dose-dependent manner after 72 h of treatment, with IC_50_ values of 2.9 µM against NF96.2 and 3.6 μM against NF2.2 (Figure 3A). In addition, LLS30 demonstrated the ability to induce apoptosis (Figure 3B). Given the frequent activation of the Ras/Erk pathway in MPNST patients, we examined whether LLS30 affects this signaling pathway. Treatment with LLS30 at 5μM for 24 h suppressed the expression of phospho-Erk in NF96.2 and NF2.2 cells (Figure 3C). Concurrently, we evaluated the efficacy of OTX008, a pre-existing Gal-1 inhibitor tested for treating advanced solid tumors in 2012 (ClinicalTrials.gov: NCT01724320), against MPNST cells. Our data revealed the limited efficacy of OTX008 against MPNST cells (Figure 3D). Taken together, LLS30 exhibits cytotoxicity against MPNST cells with superior effects compared to OTX008.

### 3.4. LLS30 Has Anti-Tumor Activity in MPNST Orthotopic Xenograft Mouse Models

**Figure 4:**
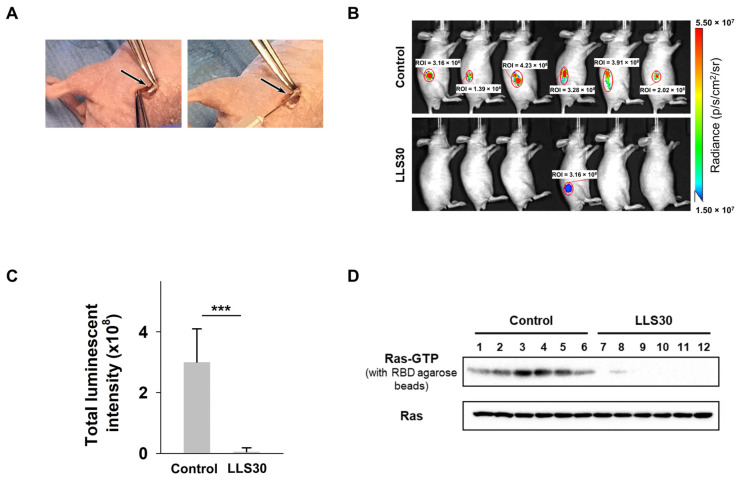
LLS30 suppresses MPNST growth in an orthotopic xenograft mouse model. (**A**) Illustration of sciatic nerve retrieval (indicated by arrows) for the injection of luciferase-tagged NF96.2 cells to establish the orthotopic xenograft mouse model. (**B**) Representative bioluminescent imaging at week 8 after LLS30 or vehicle treatment and (**C**) quantification of tumor signals. (**D**) Evaluation of activated Ras expression levels was performed using a Ras-GTP pull-down assay, followed by immunoblotting with an anti-Ras antibody (upper panel). Total Ras expression in protein extracted from both LLS30-treated and vehicle-treated tumors was assessed through immunoblotting with an anti-Ras antibody (lower panel). Samples in lanes 1–6 were treated with the vehicle, while lanes 7–12 received the LLS30 treatment. *** *p* < 0.001; two-tailed Student’s *t*-test. Data shown are mean ± s.d.

We further assessed the impact of LLS30 on MPNST growth in vivo by utilizing the orthotopic tumor model, which closely mimics the tumor microenvironment of MPNSTs. Luciferase-tagged NF96.2 cells were injected into the sciatic nerves of mice (Figure 4A). Once tumors became palpable in all mice by week 6, they were randomly assigned into two groups and intraperitoneally administered either (1) a vehicle or (2) LLS30 (10 mg/kg, once daily for 14 days). Tumor burden was monitored over time using bioluminescence imaging. As shown in Figure 4B, all six control mice exhibited significant tumor burden by week 8. In contrast, only one mouse in the LLS30-treated group exhibited a limited tumor area (Figure 4B,C). Furthermore, excised tumors were analyzed for activated Ras levels. Active Ras analysis of xenograft tumors revealed that LLS30 treatment reduced the levels of activated Ras (Figure 4D, upper panel), while total Ras protein in tumor cells remained unaffected (Figure 4D, lower panel). These studies highlight the therapeutic potential of LLS30 in reducing tumor growth in MPNST orthotopic xenograft mouse models.

### 3.5. RNA-seq Analysis Reveals the Implication of LLS30 in Various Cancer Pathways

**Figure 5:**
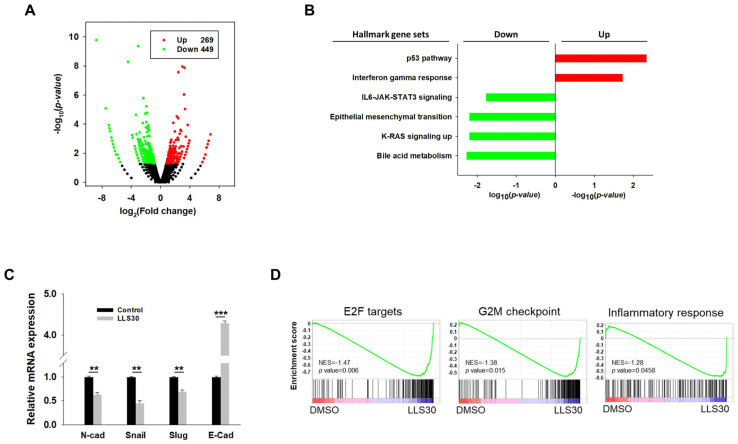
Transcriptomic analysis of LLS30 treatment in NF96.2 MPNST cells. (**A**) Volcano plot showing the log_2_ fold change (FC) and −log_10_ *p*-value for each gene, indicating expression levels and statistical significance. Each point on the plot represents an individual gene. Black points denote genes with no significant differential expression (*p* > 0.05) between the control and LLS30-treated groups, while green points represent genes that were downregulated (FC < 1.5, *p* < 0.05) and red points represent genes that were upregulated (FC > 1.5, *p* < 0.05). (**B**) Hallmark gene set analysis of the upregulated genes (FC > 1.5, *p* < 0.05) and downregulated genes (FC < 1.5, *p* < 0.05) between the control and LLS30 treatment. Pathways significantly enriched in upregulated genes are indicated by red bars, while those enriched in downregulated genes are shown by green bars. (**C**) qRT-PCR analysis of EMT markers, including N-cadherin, snail, slug, and E-cadherin, in NF96.2 cells treated with the vehicle or LLS30 (5 μM) for 24 h. (**D**) Gene set enrichment analysis (GSEA) of hallmark gene sets significantly enriched in LLS30-treated cells vs. the control, with NES indicating nominal enrichment score. A gene set shows significant enrichment at *p* < 0.05. ** *p* < 0.01, *** *p* < 0.001; two-tailed Student’s *t*-test. Data shown are mean ± s.d.

In addition to establishing that LLS30 interferes with the Gal-1/RAS interaction, we conducted a comprehensive investigation into the gene regulatory processes involved in LLS30-induced cell death. RNA-Seq data from NF96.2 cells revealed that LLS30 treatment led to the upregulation of 269 genes and the downregulation of 449 genes, each showing at least a 1.5-fold change and a *p*-value < 0.05 (Figure 5A). Functional pathway analysis of these differentially expressed genes revealed that LLS30 influences several key cellular pathways. Specifically, in the Hallmark database, upregulated genes were associated with the P53 (*p* = 4.5 × 10^−3^) and interferon gamma (IFNγ) (*p* = 1.8 × 10^−2^) pathway (Figure 5B). In addition, the downregulated pathways included bile acid metabolism, KRAS signaling, epithelial–mesenchymal transition (EMT), and IL6-JAK-STAT3 signaling (Figure 5B). qRT-PCR validated the suppression of EMT markers by LLS30, showing decreased levels of N-cadherin, snail, and slug, and increased levels of E-cadherin (Figure 5C). Furthermore, GSEA using the Hallmark gene set collection revealed that LLS30 treatment significantly disrupted proliferation-related pathways including E2F targets (*p* = 6 × 10^−3^) and the G2M checkpoint (*p* = 1.5 × 10^−2^), as well as an inflammatory response (*p* = 4.6 × 10^−2^) compared to the control group treated with DMSO (Figure 5D). These findings suggested that LLS30 not only suppresses cancer progression by regulating multiple signaling pathways but also potentially inhibits metastasis through the regulation of EMT.

### 3.6. LLS30 Inhibits Formation and Growth of Experimental MPNST Metastasis

**Figure 6:**
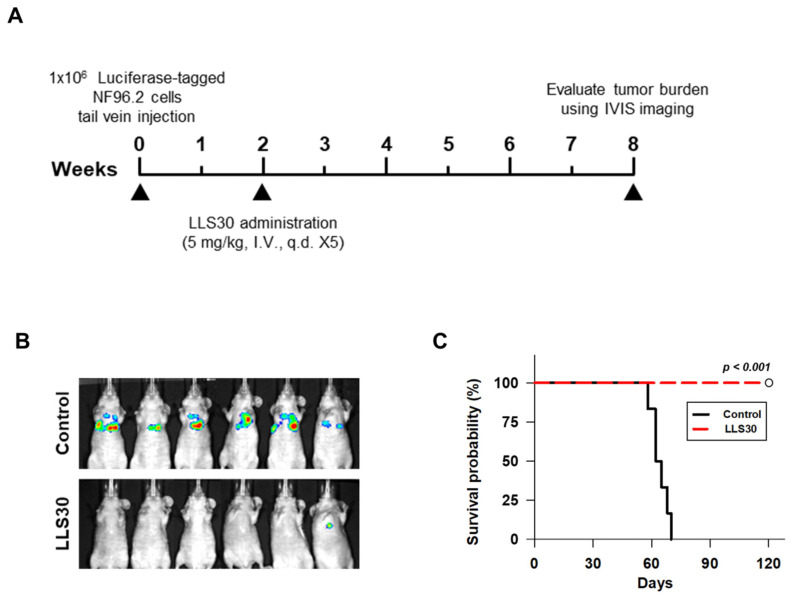
NF96.2 lung metastases-bearing mice were treated with LLS30. (**A**) The timeline illustrates the LLS30 treatment protocol. (**B**) Bioluminescent images were obtained from the control (8.7% alcohol/8.7% Tween-80) and LLS30-treated mice at 8 weeks following the initial intravenous injection of luciferase-tagged NF96.2 cells. (**C**) The Kaplan–Meier plot depicts the survival of PBS (n = 6) and LLS30-treated mice (n = 6). log-rank test.

MPNSTs, aggressive and highly metastatic sarcomas arising from the myelinating nerve sheath, often exhibit metastasis, primarily to the lung, within 2 years of initial disease presentation [26]. Noticeably, our RNA-seq analysis shows that LLS30 treatment downregulated the EMT signaling (Figure 5B,C), which is well known for its association with tumor invasion and metastasis in cancer [27]. This observation prompted us to assess whether LLS30 inhibits MPNST cell colony formation in the lung using our established experimental metastasis model [23]. Luciferase-tagged NF96.2 cells were injected into the tail vein, and lung colonies were observed two weeks after tumor cell injection. At this point, LLS30 was administered at a lower dose of 5 mg/kg to mitigate its effect on cell proliferation, with the treatment duration spanning five days. By week 8, macroscopic lung metastases were evident in all six control mice (Figure 6B). Conversely, among the LLS30-treated group, no visible metastases were detected in five mice, while one exhibited a slight signal (Figure 6B). Importantly, LLS30 treatment significantly improved survival (*p* < 0.001 compared to the control; Figure 6C). While all control mice had died by day 70, all LLS30-treated mice remained alive at 120 days. These results demonstrate that LLS30 treatment inhibits the growth of MPNST lung metastases, leading to a significant increase in survival.

## 4. Discussion

The pivotal role of RAS in malignancies makes them the primary targets for cancer therapies. Despite extensive efforts, attempts to target RAS and develop clinically approved drugs have proven unsuccessful. Given that RAS proteins require membrane association for their biological activity, disrupting this association emerges as a viable strategy for cancer treatment. In this study, we confirmed the role of LLS30 as a Gal-1 inhibitor for treating MPNSTs, demonstrating that LLS30 disrupts the Gal-1/Ras interaction, leading to Ras dissociation from the plasma membrane and subsequent suppression of Ras activation. Our study provides valuable insights into the molecular mechanisms underlying the anti-cancer effects of LLS30 and highlights its promise as a therapeutic agent for MPNST treatment.

Significant research has explored Gal-1 inhibition in experimental cancer models. Thiodigalactoside has shown efficacy in slowing breast cancer progression when combined with vaccine immunotherapy [28,29,30,31]. OTX008, a potent small molecule inhibitor of Gal-1, interferes with ERK signaling and causes G2/M cell cycle arrest in multiple human cancer cell lines [32]. Preclinical studies have indicated that OTX008 shows promising efficacy against solid tumors, either as a monotherapy or in combination with standard treatments [33,34]. Despite a 2012 clinical trial evaluating OTX008 for advanced solid tumors, its outcome remains undisclosed. This underscores the limited effectiveness of existing Gal-1 inhibitors for human use and the urgent need for safer and more effective alternatives. In this study, preclinical evidence showed that LLS30 inhibited tumor growth and metastasis in MPNST mouse models. To the best of our knowledge, our team is currently the sole entity examining Gal-1 inhibition through LLS30 specifically in the context of MPNSTs. Our findings expand the scope of Gal-1 inhibitors, paving the way for novel therapeutic strategies in the management of MPNSTs.

Growing evidence indicates that tumor-intrinsic signaling can modulate the immune response to the tumor [35]. Tumors exhibiting tumor-infiltrating lymphocytes (TILs), PD-L1 expression, tumor-associated immune cells, potential genomic instability, and a pre-existing antitumoral immune response (“hot” tumors) generally show better responsiveness to immunotherapy. In contrast, “cold” tumors, which lack these features, generally require treatments that transform them into “hot” tumors to improve their response to immunotherapy. MPNSTs are classified as immunologically “cold” tumors, characterized by low levels of T cell infiltration [36,37]. In this study, following LLS30 treatment, we observed the upregulation of IFNγ, a potent switch and enhancer for the recruitment of innate immune cells. This exciting result suggested that LLS30 may have the potential to transform immunologically “cold” tumors into “hot” ones by upregulating IFNγ. Consequently, this could increase TILs and enhance the MPNST response to PD-1 checkpoint inhibitor therapies. However, there have been no published in vivo studies exploring immunotherapy regimens tailored specifically to MPNSTs. We are currently actively investigating immunotherapy for MPNSTs, specifically focusing on examining the combined effects of LLS30 and PD-1 checkpoint inhibitors for treatment.

EMT is well established as a pivotal mechanism driving tumor invasion and metastasis in MPNSTs. Studies showed that loss of the tumor suppressor protein tyrosine phosphatase receptor S (PTPRS) promotes EMT in MPNSTs by increasing the expression of markers like N-cadherin and αSMA while reducing E-cadherin, ultimately enhancing cellular motility and invasiveness [38]. Consistent with EMT’s role in cancer progression, Gal-1 has been implicated in facilitating EMT across various cancers, such as hepatocellular carcinoma and ovarian cancer, through activation of pathways like PI3K/AKT and MAPK JNK/p38 [39,40]. Targeting the Gal-1 function is a rational approach to potentially attenuate the metastatic capacity of MPNST cells. Indeed, our results showed that Gal-1 suppression through LLS30 decreased levels of EMT markers resulting in reduced metastasis in MPNSTs. However, the detailed mechanisms by which Gal-1 mediates EMT in MPNSTs remain unclear. Further investigation is warranted to elucidate these pathways.

Our findings establish the therapeutic potential of targeting the Gal-1/Ras interaction for MPNST treatment, with LLS30 emerging as a promising agent. LLS30 not only inhibits Ras-driven tumor growth but also affects epithelial–mesenchymal transition (EMT), thereby suppressing metastasis, a critical aspect of MPNST pathology. This dual mechanism highlights the value of Gal-1 inhibition in treating aggressive cancers like MPNSTs and opens new avenues for therapeutic development. Future studies could incorporate patient-derived biopsies or iPS cells to better model the complexity and microenvironment of MPNSTs, which would enhance the understanding of LLS30’s therapeutic potential and clinical applicability. These models would also address MPNST heterogeneity, refining therapeutic strategies. Although traditional docking methods were employed in this study, future iterations could benefit from incorporating transformer models, such as those explored in recent studies [41,42]. IND-enabling studies are essential to assess pharmacokinetics (PK), toxicity, formulation feasibility, and stability in preparation for clinical trials. These studies will provide critical information on how LLS30 behaves in vivo and its potential for safe clinical application. In addition, combination therapy trials, including LLS30 with other treatments (e.g., PD-1 checkpoint inhibitors or chemotherapy), will be considered to enhance therapeutic efficacy and explore potential synergistic effects.

## 5. Conclusions

This study demonstrates that LLS30 holds significant therapeutic potential in MPNST treatment by disrupting the Gal-1/Ras interaction and targeting pathways involved in tumor growth and metastasis. These results encourage further research and clinical trials to assess the broader applicability of Gal-1 inhibition as a viable treatment for MPNSTs and potentially other aggressive cancers.

## Figures and Tables

**Table 1:** The contact list between Gal-1 with H-Ras(G12V).

Chain 1	Residue	Chain 2	Residue	Interaction Type
H-Ras(G12V)	Gln25.CA	Gal-1	Cys2.SG	Hydrogen bond interaction
H-Ras(G12V)	Tyr32.OH	Gal-1	Asp123.OD1	Hydrogen bond interaction
H-Ras(G12V)	Thr35.OG1	Gal-1	Asn33.OD1	Hydrogen bond interaction
H-Ras(G12V)	Glu37.OE1	Gal-1	His52.ND1	Salt bridge
H-Ras(G12V)	Asp38.OD1	Gal-1	Ser29.OG	Hydrogen bond interaction
H-Ras(G12V)	Asp38.OD1	Gal-1	His52.NE2	Salt bridge
H-Ras(G12V)	Tyr40.OH	Gal-1	Asp123.OD1	Hydrogen bond interaction
H-Ras(G12V)	Arg41.NH2	Gal-1	Asp26.OD2	Salt bridge
H-Ras(G12V)	Glu63.OE1/OE2	Gal-1	Lys63.NZ	Salt bridge
H-Ras(G12V)	Glu63.OE1	Gal-1	Ala67.CA	Hydrogen bond interaction
H-Ras(G12V)	Glu63.OE2	Gal-1	Trp68.NE1	Hydrogen bond interaction
H-Ras(G12V)	Met67.N	Gal-1	His52.O	Hydrogen bond interaction

## Data Availability

Data are available upon request.

## Abbreviations

Gal-1: Galectin-1; MPNST: malignant peripheral nerve sheath tumor; NF1: neurofibromatosis Type 1; IFNγ: interferon gamma; TILs: tumor-infiltrating lymphocytes; and PD-L1: Programmed Death-Ligand 1.
